# Dopamine-Derived Oxidative Stress in Attention-Deficit/Hyperactivity Disorder: A Narrative Review of Molecular Mechanisms, Neural Circuitry, and Therapeutic Implications

**DOI:** 10.3390/antiox15050613

**Published:** 2026-05-13

**Authors:** George Țocu, Bogdan Ioan Ștefănescu, Lavinia Țocu, Florentin Dimofte, Valerii Luțenco, Loredana Stavăr Matei, Marius Dumitru Dănilă, Mihaela Cristina Marin, Mădălina Nicoleta Matei, Oana Mariana Mihailov, Paul Iacobescu, Raul Mihailov

**Affiliations:** 1 Faculty of Medicine and Pharmacy, Research Center in the Medical-Pharmaceutical Field, “Dunărea de Jos” University, 800008 Galati, Romania; george.tocu@ugal.ro (G.Ț.); florentin.dimofte@ugal.ro (F.D.); valerii.lutenco@ugal.ro (V.L.); loredana.matei@ugal.ro (L.S.M.); marius.danila@ugal.ro (M.D.D.); cristina.marin@ugal.ro (M.C.M.); madalina.matei@ugal.ro (M.N.M.); oana.mihailov@ugal.ro (O.M.M.); raul.mihailov@ugal.ro (R.M.); 2 “Sf. Apostol Andrei” County Emergency Clinical Hospital, 800578 Galati, Romania; 3 “Sf. Ioan” Children’s Emergency Hospital, 800487 Galati, Romania; 4 “Sfântul Spiridon” Clinical Hospital of Pneumophthisiology, 800552 Galati, Romania; 5 Computer Science and Information Technology Department, “Dunărea de Jos” University, 800008 Galati, Romania; paul.iacobescu@ugal.ro

**Keywords:** ADHD, dopamine, oxidative stress, reactive oxygen species, mitochondrial dysfunction, dopaminergic circuits

## Abstract

Attention-deficit/hyperactivity disorder (ADHD) is a common neurodevelopmental disorder in which dopaminergic dysfunction plays a central role. Beyond its neurotransmitter function, dopamine is a redox-active molecule capable of generating reactive oxygen species and toxic intermediates, particularly when cytosolic dopamine accumulates because of altered vesicular storage or transporter imbalance. This review examines whether dopamine-derived oxidative stress may represent a biologically plausible and testable framework for ADHD by integrating current evidence on dopamine metabolism, oxidative stress, and neuronal dysfunction, while distinguishing direct evidence from indirect and translational findings. A structured literature search was conducted in PubMed, Scopus, and Web of Science for relevant English-language studies published between January 2000 and March 2026. The available evidence suggests that dopamine-derived oxidative stress may help link disturbed dopamine handling to protein modification, lipid peroxidation, mitochondrial dysfunction, synaptic inefficiency, and circuit-level abnormalities in ADHD. Although direct in vivo evidence remains limited, this framework may help distinguish dopamine-derived oxidative stress from more general oxidative imbalance in ADHD and may guide future biomarker-based, experimental, and translational research.

## 1. Introduction

Attention-deficit/hyperactivity disorder (ADHD) is one of the most common neurodevelopmental disorders, characterized by inattention, impulsivity, and hyperactivity [1]. Although its etiopathogenesis is multifactorial, involving genetic, neurodevelopmental, and environmental determinants, dysfunction of the dopaminergic system remains a central component of current neurobiological models [2]. Altered dopaminergic neurotransmission, particularly within frontostriatal and mesocorticolimbic circuits, has been consistently associated with the clinical phenotype of ADHD, suggesting that dysregulation of dopamine homeostasis plays a key role in disease pathogenesis [3].

Beyond its classical role as a neurotransmitter, dopamine is an intrinsically redox-unstable molecule. Its catecholic structure predisposes it to auto-oxidation and enzymatic metabolism that generate reactive oxygen species (ROS), including superoxide anion (O_2_^•−^), hydrogen peroxide (H_2_O_2_), and hydroxyl radical (^•^OH) [4]. Under conditions in which cytosolic dopamine exceeds the capacity of neuronal storage and detoxification systems, it becomes an important endogenous source of oxidative stress [5]. This concept, referred to as “dopamine-derived oxidative stress,” provides a mechanistic framework for understanding the vulnerability of dopaminergic neurons.

Dopamine contributes to ROS generation through multiple interconnected pathways. Auto-oxidation leads to the formation of reactive semiquinones and quinones, accompanied by O_2_^•−^ and H_2_O_2_ production [6]. In parallel, monoamine oxidase (MAO)-mediated metabolism generates H_2_O_2_ and reactive aldehydes such as 3,4-dihydroxyphenylacetaldehyde (DOPAL), a metabolite with significant neurotoxic potential [7]. Additionally, interactions with transition metals, such as iron and copper, can amplify ROS production via Fenton-type reactions, promoting a pro-oxidative intracellular environment that affects proteins, lipids, and DNA [8].

While these mechanisms have been extensively characterized in neurodegenerative disorders, particularly in Parkinson’s disease, their relevance in ADHD remains comparatively underexplored. Most mechanistic data currently derive from experimental dopaminergic models and neurodegenerative paradigms, whereas ADHD-specific evidence remains more limited and largely indirect. Available studies in ADHD have primarily focused on dopaminergic dysfunction, oxidative stress markers, and emerging alterations in dopamine transporter (DAT) and vesicular monoamine transporter 2 (VMAT2) balance, without fully integrating these processes into a unified mechanistic framework. This gap in knowledge supports the need to examine dopamine-derived oxidative stress within the specific neurobiological context of ADHD.

In ADHD, these mechanisms gain particular relevance. Neurobiological models suggest an imbalance between dopamine release, reuptake, and storage, favoring an increased cytosolic fraction, the form most susceptible to oxidation [9]. Thus, not only dopamine levels per se are important, but also their intracellular handling. This perspective supports the hypothesis that dopamine-derived oxidative stress may act as an intermediate pathogenic mechanism linking dopaminergic dysfunction to the structural and functional alterations observed in ADHD-related neural circuits, particularly in the prefrontal cortex and striatum [10].

Despite growing evidence on oxidative stress in ADHD, most studies address this process in a nonspecific manner, without distinguishing ROS sources. This limitation hinders mechanistic understanding and the development of targeted therapies. Therefore, focusing specifically on dopamine-derived oxidative stress may provide a more refined conceptual approach and open new directions for translational research.

The aim of this review is to integrate current evidence on dopamine metabolism, oxidative stress, and neuronal dysfunction in order to propose a biologically plausible and testable framework for ADHD, while distinguishing direct evidence from indirect and translational findings. More specifically, the review seeks to distinguish dopamine-derived oxidative stress from more general oxidative imbalance in ADHD, to integrate molecular and circuit-level findings, and to highlight the major translational gaps that currently limit direct clinical application.

## 2. Methods

This article was designed as a narrative review focused on the mechanistic analysis of dopamine-derived oxidative stress in ADHD, integrating evidence from biochemical, neurobiological, genetic, experimental, and clinical studies. The methodological approach was intended to provide a critical and coherent synthesis of the available literature, with emphasis on mechanistic plausibility and translational relevance. Because direct mechanistic evidence in ADHD remains limited, the reviewed literature was interpreted across three levels of relevance: direct evidence from human ADHD studies, indirect evidence from ADHD-relevant experimental or clinical models, and broader dopaminergic mechanistic evidence when directly informative for the conceptual framework proposed in this review.

A structured literature search was conducted in PubMed/MEDLINE, Scopus, and Web of Science to identify relevant studies addressing dopamine metabolism, oxidative stress, and their potential role in ADHD. The search strategy was based on combinations of keywords and Boolean operators, including “ADHD”, “dopamine”, “oxidative stress”, “reactive oxygen species”, “dopamine oxidation”, “dopamine quinone”, “monoamine oxidase”, “DOPAL”, “VMAT2”, “dopamine transporter”, “dopaminergic dysfunction”, “neurodevelopment”, “prefrontal cortex”, “striatal circuitry”, “mitochondrial dysfunction”, and “ferroptosis”. Only English-language articles published between January 2000 and March 2026 were considered. Priority was given to recent studies, while older seminal articles were retained when relevant to fundamental biochemical and mechanistic concepts.

The review included original experimental studies, both in vitro and in vivo, investigating dopamine oxidation, ROS generation, vesicular dopamine handling, monoamine oxidase-related pathways, mitochondrial dysfunction, ferroptosis-related mechanisms, and redox-dependent neuronal injury. Clinical and observational studies evaluating oxidative stress in ADHD were also considered, together with neuroimaging studies relevant to dopaminergic circuit dysfunction, genetic studies involving key regulators of dopamine metabolism or transport, including DAT1, COMT, MAO, and VMAT2, and selected review articles with clear mechanistic relevance. Given the limited direct ADHD-specific literature, both ADHD-focused studies and mechanistically relevant evidence from broader dopaminergic research were considered. Reference lists of key articles were also screened manually to identify additional studies.

Studies were excluded if they addressed oxidative stress without a direct or plausible link to dopamine metabolism, lacked sufficient methodological clarity, provided limited mechanistic value, or were redundant in relation to more informative publications. The selected literature was analyzed using an integrative approach focused on linking dopamine oxidation pathways with their molecular, cellular, and circuit-level consequences, while distinguishing dopamine-derived oxidative stress from more general redox abnormalities in order to preserve the conceptual focus of the review.

## 3. Dopamine Metabolism as a Primary Source of Reactive Oxygen Species

### 3.1. Reactive Oxygen Species Generated from Dopamine Metabolism

Dopamine oxidation represents a major endogenous source of reactive oxygen species (ROS), generating a spectrum of chemically distinct intermediates with different reactivity, stability, and biological impact. The primary ROS formed during dopamine metabolism include O_2_^•−^, H_2_O_2_, and ^•^OH, together with secondary lipid-derived radicals produced during downstream oxidative cascades [11,12].

O_2_^•−^ arises during dopamine auto-oxidation and quinone redox cycling, primarily through one-electron transfer reactions involving semiquinone intermediates. Although relatively short-lived, it occupies a central position in oxidative cascades, serving as the immediate precursor of H_2_O_2_ [13]. Through spontaneous or enzymatically catalyzed dismutation, O_2_^•−^ is rapidly converted into H_2_O_2_, thereby linking localized redox reactions to more diffusible oxidative stress [11].

**Figure 1:**
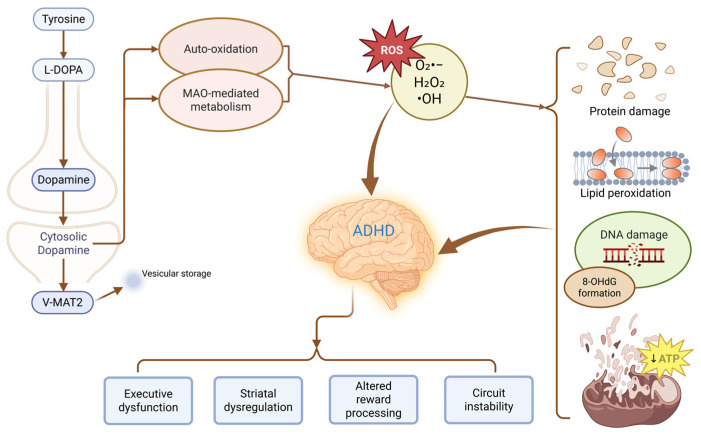
Dopamine-derived oxidative stress pathways and their impact on neuronal dysfunction in ADHD. Dopamine is synthesized from tyrosine via L-3,4-dihydroxyphenylalanine (L-DOPA) and transiently exists in the cytosol before being sequestered into synaptic vesicles through vesicular monoamine transporter 2 (VMAT2), a process that limits oxidation-prone cytosolic exposure. When cytosolic dopamine undergoes auto-oxidation and monoamine oxidase (MAO)-mediated metabolism, reactive oxygen species (ROS), including O_2_^•−^, H_2_O_2_, and ^•^OH, are generated. These oxidative processes contribute to protein damage, lipid peroxidation, DNA damage, including 8-hydroxy-2′-deoxyguanosine (8-OHdG) formation, and mitochondrial dysfunction associated with reduced ATP availability. At the systems level, these molecular alterations may disrupt dopaminergic circuit function, potentially contributing to executive dysfunction, striatal dysregulation, altered reward processing, and broader circuit instability relevant to ADHDCreated in BioRender. Ciobarceanu, G. (2026) https://BioRender.com/vmrfyto (accessed on 7 May 2026).

H_2_O_2_ is generated both from superoxide dismutation and directly through MAO-mediated dopamine metabolism. Compared to O_2_^•−^, H_2_O_2_ is relatively stable and membrane-permeable, allowing it to diffuse across intracellular compartments, including mitochondria and the nucleus [14]. While it participates in physiological redox signaling, its accumulation promotes oxidative stress and provides the substrate for Fenton-type reactions, leading to the formation of ^•^OH, which, produced in the presence of transition metals such as ferrous iron, represents the most reactive ROS derived from dopamine metabolism; its extreme reactivity results in rapid and non-specific damage to proteins, lipids, and nucleic acids, and because no enzymatic detoxification systems exist for ^•^OH, its biological effects are determined primarily by the microenvironment in which it is generated [15]. Figure 1 illustrates the generation and interplay of reactive oxygen species arising from dopamine metabolism.

Beyond these primary ROS, dopamine oxidation also initiates lipid peroxidation cascades, leading to the formation of lipid peroxyl radicals and reactive aldehydes such as 4-hydroxynonenal. In parallel, dopamine-derived quinones can impair antioxidant defense systems. A recent study showed that dopamine oxidation promotes degradation of glutathione peroxidase 4, thereby reducing lipid peroxide detoxification capacity and increasing susceptibility to ferroptotic pathways. Together, these processes establish dopamine metabolism as a central driver of intracellular oxidative burden [16].

### 3.2. Dopamine Synthesis, Compartmentalization, and Cytosolic Vulnerability

Dopamine homeostasis depends not only on synthesis, but also on strict intracellular compartmentalization. Dopamine is synthesized in the cytosol from tyrosine and must be rapidly sequestered into synaptic vesicles by VMAT2. This sequestration represents a critical redox-protective mechanism, as it limits the exposure of free dopamine to the cytosolic environment, where oxidation and the formation of O_2_^•−^ and H_2_O_2_ are favored.

Vesicular storage occurs in an acidic lumen, which stabilizes dopamine and reduces its oxidation potential. Structural studies have demonstrated that VMAT2 uses the vesicular proton gradient, exchanging luminal protons for cytosolic monoamines, thereby maintaining dopamine in a chemically protected environment. A recent study further clarified this mechanism and reinforced the concept that vesicular acidification is central to limiting dopamine oxidation [17].

The pathological relevance of cytosolic dopamine has been demonstrated experimentally. A study using intracellular electrochemical techniques quantified cytosolic dopamine in dopaminergic neurons and showed that increased intracellular dopamine levels are directly associated with enhanced oxidative stress and neuronal vulnerability [18]. These findings support the concept that toxicity is driven not by total dopamine content, but specifically by the non-sequestered cytosolic fraction, which is prone to generating O_2_^•−^ and H_2_O_2_.

Additional experimental models have shown that disruption of dopamine compartmentalization is sufficient to induce oxidative neurodegeneration. One study demonstrated that neurons exposed to unregulated cytosolic dopamine develop progressive oxidative damage, including protein oxidation and glutathione depletion [19]. Other work has shown that vesicular dysfunction increases cytosolic catecholamine levels and promotes oxidative stress, while more recent evidence indicates that impaired VMAT2 function directly enhances dopamine oxidation. Collectively, these findings establish cytosolic dopamine as a critical upstream source of ROS generation [20].

In the context of ADHD, this mechanism is relevant because abnormalities in dopamine handling, rather than dopamine concentration alone, may influence the size of the cytosolic dopamine pool and thereby affect oxidative vulnerability in dopamine-sensitive circuits.

### 3.3. Dopamine Auto-Oxidation and Quinone Formation

Dopamine can undergo spontaneous auto-oxidation under physiologically relevant conditions, producing semiquinone radicals, dopamine quinones, O_2_^•−^, and H_2_O_2_. This process is strongly influenced by environmental factors, particularly pH, oxygen availability, and dopamine concentration [21].

Under near-neutral cytosolic conditions, dopamine oxidation is favored, whereas acidic environments markedly suppress this process. A mechanistic study demonstrated that acidic pH significantly slows dopamine auto-oxidation, supporting the concept that vesicular storage protects against ROS formation, while cytosolic exposure promotes O_2_^•−^ and H_2_O_2_ generation [13].

The quinones formed during dopamine oxidation are highly reactive electrophiles that covalently modify proteins and disrupt cellular function, including mitochondrial activity. Human brain studies have confirmed the presence of cysteinyl-dopamine conjugates, which serve as in vivo markers of dopamine oxidation and quinone formation, providing direct evidence that these processes occur physiologically [22].

More recent work has shown that dopamine oxidation can progress beyond initial quinone formation. A study demonstrated that under Fenton-like conditions, dopamine undergoes sequential oxidation to highly reactive derivatives, with H_2_O_2_ and ^•^OH participating in these transformations. These findings indicate that dopamine oxidation is not a single-step process, but an evolving cascade that generates increasingly reactive intermediates [23].

### 3.4. Monoamine Oxidase-Mediated Metabolism

Monoamine oxidase provides a major enzymatic pathway linking dopamine metabolism to oxidative stress. MAO-A and MAO-B, located on the outer mitochondrial membrane, catalyze the oxidative deamination of dopamine, producing H_2_O_2_ and the reactive DOPAL. H_2_O_2_ generated through MAO activity contributes directly to oxidative stress and can be converted into ^•^OH in the presence of redox-active metals. In parallel, DOPAL acts as a highly reactive catecholaldehyde capable of modifying proteins and amplifying oxidative injury [24].

Importantly, MAO-derived ROS production is spatially organized. A study demonstrated that dopamine metabolism through MAO is coupled to mitochondrial processes, resulting in localized oxidative stress within mitochondria rather than diffuse cytosolic accumulation of H_2_O_2_ [14]. This compartmentalization suggests that MAO activity contributes to targeted mitochondrial redox imbalance.

Further evidence indicates that dopamine oxidation can impair antioxidant defenses downstream. A recent study showed that dopamine oxidation promotes degradation of glutathione peroxidase 4, linking dopamine metabolism not only to ROS generation, including H_2_O_2_ and secondary ^•^OH formation, but also to reduced cellular capacity to counteract oxidative damage [25]. This establishes MAO-mediated metabolism as both a direct and indirect contributor to oxidative stress.

Although demonstrated mainly outside ADHD-specific models, this pathway is pertinent to ADHD because altered dopamine turnover may increase the relative contribution of MAO-mediated metabolism to intracellular oxidative burden, particularly in metabolically active dopaminergic networks.

### 3.5. Dopamine-Derived Redox Cycling

Following initial oxidation, dopamine-derived quinones can undergo redox cycling, a process that transforms transient oxidative events into sustained ROS production. In this cycle, quinones are reduced to semiquinones, which subsequently transfer electrons to molecular oxygen, regenerating the oxidized quinone while producing O_2_^•−^. This leads to continuous formation of O_2_^•−^ and secondary accumulation of H_2_O_2_ [26]. The persistence of this cycle depends on intracellular reducing equivalents such as NADH and NADPH, which sustain the regeneration of semiquinone intermediates. As a result, dopamine oxidation becomes a self-propagating process, amplifying oxidative stress beyond the initial trigger [27].

Experimental studies have shown that dopamine oxidation products form covalent adducts with cysteine-containing proteins and deplete glutathione, indicating that thiol systems play a central role in modulating quinone reactivity. Additional evidence demonstrates that glutathione and cysteine can intercept quinone intermediates, limiting redox cycling and preventing progression toward more oxidized species [23].

Human brain data further support the relevance of these mechanisms in vivo, with the detection of cysteinyl-dopamine conjugates indicating ongoing quinone chemistry in dopaminergic regions [22]. At the same time, recent findings show that dopamine oxidation can impair antioxidant systems, including the loss of GPX4, thereby enhancing lipid peroxidation and reinforcing the coupling between O_2_^•−^ production, H_2_O_2_ accumulation, and cellular vulnerability [16].

### 3.6. Metal-Catalyzed Dopamine Oxidation

Transition metals, particularly iron and copper, play a critical role in amplifying dopamine-derived oxidative stress by modulating both the rate and outcome of dopamine oxidation reactions. These metals facilitate electron transfer processes that promote quinone formation and enhance ROS generation [27].

H_2_O_2_ generated during dopamine metabolism is converted through Fenton reactions into ^•^OH, linking dopamine turnover directly to the formation of the most reactive ROS. This interaction makes metal availability a key determinant of oxidative stress intensity [16].

Experimental work has demonstrated that iron and copper not only accelerate dopamine oxidation, but also modify the structure of downstream products, including melanin-like polymers. These findings indicate that metal-catalyzed reactions influence not only reaction kinetics, but also the biochemical fate of dopamine [15].

More recent studies have shown that iron-dependent Fenton systems enable sequential oxidation steps leading to highly reactive quinone derivatives, further expanding the spectrum of oxidative intermediates. Together with earlier kinetic data demonstrating the influence of pH, these observations indicate that dopamine oxidation is highly context-dependent, shaped by local chemical conditions [23].

Overall, transition metals amplify dopamine-derived oxidative stress by increasing both the rate of ROS generation, including ^•^OH, and the diversity of reactive intermediates produced, thereby intensifying cellular oxidative injury.

The relevance to ADHD lies not in disease-specific proof of metal-driven dopamine oxidation, which remains limited, but in the possibility that local redox conditions may amplify the consequences of abnormal dopamine handling in vulnerable neural circuits.

## 4. Molecular Consequences of Dopamine-Derived Reactive Oxygen Species

### 4.1. Protein Modification and Enzyme Dysfunction

Dopamine-derived oxidative species, particularly dopamine quinones, profoundly affect protein structure and function through covalent post-translational modifications. As highly electrophilic intermediates, dopamine quinones react preferentially with nucleophilic amino acid residues, especially cysteine thiols, forming stable dopamine–protein adducts [28]. Biochemical and proteomic studies have shown that these reactions induce widespread protein modification, leading to conformational alterations, loss of enzymatic activity, and increased aggregation propensity [29].

Experimental evidence has identified multiple intracellular targets of dopamine quinone. In rat brain mitochondria, proteomic analyses demonstrated covalent modification of key mitochondrial proteins, including components of the electron transport chain, chaperones, and metabolic enzymes, resulting in impaired mitochondrial function and altered energy metabolism [30]. Proteins involved in cellular stress responses, such as DJ-1 and ubiquitin-related enzymes, were also found to be affected, indicating that dopamine oxidation disrupts not only basal cellular processes but also adaptive protective pathways [31].

A particularly important example is glutathione peroxidase 4 (GPX4), a key mitochondrial antioxidant enzyme. Hauser et al. showed that dopamine quinone directly binds to GPX4, reducing its abundance and enzymatic activity. In both isolated mitochondria and neuronal models, dopamine exposure induced GPX4 degradation and polymerization, thereby impairing lipid peroxide detoxification [25]. More recent work further demonstrated that dopamine oxidation promotes GPX4 ubiquitination and degradation in dopaminergic neurons, suggesting that dopamine-derived oxidative stress alters not only protein structure but also protein turnover [16].

Collectively, these findings indicate that dopamine-derived quinones are potent modifiers of the neuronal proteome, compromising enzymatic activity, mitochondrial integrity, and antioxidant defenses. In the context of ADHD, the relevance of these processes lies in the possibility that such protein modifications may contribute to subtle but functionally important disturbances in synaptic signaling, cellular stress responses, and neuronal resilience, even in the absence of overt neurodegeneration.

### 4.2. Lipid Peroxidation

Lipid membranes are highly susceptible to oxidative damage due to their high content of polyunsaturated fatty acids, which can undergo peroxidation in the presence of reactive oxygen species. Dopamine-derived ROS, including O_2_^•−^, H_2_O_2_, and secondary radicals generated via metal-catalyzed reactions, initiate lipid peroxidation cascades that compromise membrane integrity and cellular function [8,32].

Although dopamine itself does not directly target lipids, its oxidation products indirectly drive lipid peroxidation through ROS generation and depletion of antioxidant defenses. A critical link between dopamine oxidation and lipid peroxidation has been established through studies on GPX4, an enzyme responsible for reducing phospholipid hydroperoxides. As demonstrated by Hauser et al., dopamine quinone-mediated inactivation of GPX4 results in impaired detoxification of lipid peroxides, thereby facilitating the accumulation of peroxidized lipids [25].

This mechanism was further refined in recent work showing that dopamine oxidation promotes ferroptosis, a form of regulated cell death characterized by lipid peroxidation. In dopaminergic neurons, dopamine oxidation was shown to trigger GPX4 ubiquitination and degradation, leading to unchecked lipid peroxidation and neuronal death [16]. These findings provide strong experimental support for a close link between dopamine-derived oxidative stress and lipid membrane damage through both ROS generation and impairment of lipid repair systems.

In addition, the interaction between dopamine-derived quinones and membrane-associated proteins may further destabilize lipid bilayers. Covalent modification of membrane proteins can alter membrane fluidity, disrupt ion channel function, and impair vesicular trafficking, thereby amplifying the functional consequences of lipid peroxidation [33].

Thus, lipid peroxidation in dopaminergic neurons should be understood not merely as a downstream effect of oxidative stress, but as a process tightly linked to dopamine metabolism through both direct and indirect mechanisms. While direct evidence in ADHD remains limited, this pathway may be relevant to the disorder because lipid membrane integrity is essential for receptor function, vesicular trafficking, and synaptic efficiency in circuits repeatedly implicated in ADHD.

### 4.3. DNA and Mitochondrial Damage

Dopamine-derived reactive oxygen species can induce significant damage to both nuclear and mitochondrial DNA, contributing to genomic instability and impaired cellular function. Reactive species such as hydroxyl radicals are capable of inducing base modifications, strand breaks, and cross-linking, with 8-hydroxy-2′-deoxyguanosine (8-OHdG) representing one of the most widely studied markers of oxidative DNA damage [34,35].

Experimental models have demonstrated that increased cytosolic dopamine and impaired vesicular storage lead to elevated oxidative stress and DNA damage. In VMAT2-deficient models, reduced dopamine sequestration results in increased oxidative burden and alterations in DNA repair pathways, indicating that dopamine-derived ROS directly affect genomic maintenance mechanisms. These findings suggest that DNA damage is not merely a consequence of generalized oxidative stress, but is specifically linked to dysregulated dopamine handling [36].

Mitochondrial DNA is particularly vulnerable due to its proximity to the electron transport chain and limited repair capacity. Proteomic and biochemical studies have shown that dopamine oxidation products impair mitochondrial function, leading to increased ROS production and further DNA damage [37]. This creates a vicious cycle in which mitochondrial dysfunction amplifies oxidative stress, further damaging mitochondrial DNA and impairing cellular energetics.

Moreover, dopamine-derived quinones can directly modify mitochondrial proteins involved in DNA maintenance and replication, further compromising mitochondrial integrity [38]. Given the central role of mitochondria in neuronal survival, especially in energy-demanding dopaminergic neurons, these effects have significant implications for neuronal function and viability.

### 4.4. Dopamine-Specific Mitochondrial Toxicity

Mitochondria represent a primary target of dopamine-derived oxidative stress due to their dual role as both sources and targets of reactive oxygen species. Dopamine oxidation products, including quinones and reactive aldehydes such as DOPAL, interact with mitochondrial proteins, leading to dysfunction of the electron transport chain, impaired ATP production, and increased ROS generation [39].

Proteomic analyses have identified multiple mitochondrial proteins that are susceptible to modification by dopamine quinones, including components of complexes I and III, as well as enzymes involved in energy metabolism [30]. These modifications result in decreased enzymatic activity and altered mitochondrial respiration, contributing to energy deficits in dopaminergic neurons.

The impact of dopamine oxidation on mitochondrial antioxidant systems is also significant. As previously discussed, GPX4 is a critical enzyme for maintaining mitochondrial redox balance, and its modification or degradation leads to increased susceptibility to oxidative damage [16,25]. In addition, dopamine-derived ROS can impair other antioxidant systems, including glutathione metabolism, further exacerbating mitochondrial dysfunction [16,40].

**Figure 2:**
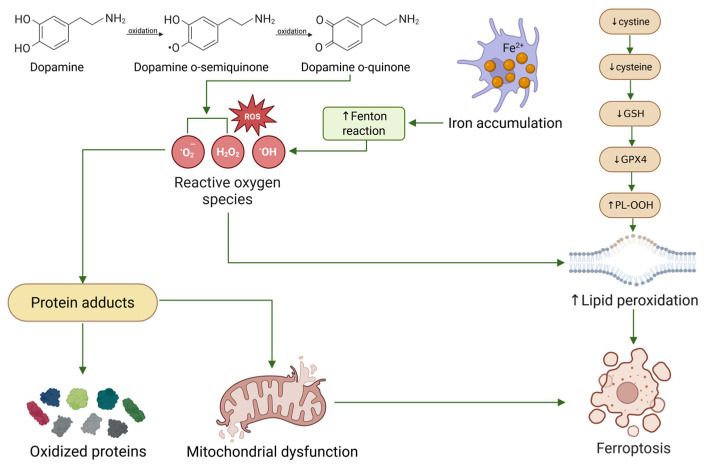
Molecular pathways linking dopamine oxidation to reactive oxygen species generation, mitochondrial dysfunction, and ferroptosis. Dopamine oxidation generates semiquinone and quinone intermediates that stimulate the production of reactive oxygen species (ROS), particularly O_2_^•−^ and H_2_O_2_, while intracellular iron accumulation enhances Fenton-driven oxidative reactions leading to ^•^OH generation and intensified oxidative membrane damage. These reactive species promote protein adduct formation, oxidative protein damage, and mitochondrial dysfunction, while also increasing cellular vulnerability to ferroptotic processes. In parallel, reduced cystine uptake, decreased cysteine availability, reduced glutathione (GSH) synthesis, and diminished glutathione peroxidase 4 (GPX4) activity impair the detoxification of phospholipid hydroperoxides, leading to phospholipid hydroperoxide (PL-OOH) accumulation and enhanced lipid peroxidation. Collectively, these interconnected mechanisms illustrate a molecular framework linking dopamine oxidation, redox imbalance, mitochondrial injury, and ferroptosis-related neuronal vulnerability, potentially contributing to the oxidative and neurobiological disturbances implicated in ADHD. Created in BioRender. Ciobarceanu, G. (2026) https://BioRender.com/k80tqxw (accessed on 7 May 2026).

Recent studies have also highlighted the role of dopamine oxidation in activating cell death pathways linked to mitochondrial dysfunction [16,40]. One such mechanism may involve ferroptosis, in which depletion of GSH and GPX4 weakens antioxidant defense, impairs phospholipid hydroperoxide detoxification, and promotes PL-OOH accumulation and lipid peroxidation [16,40]. Experimental findings further suggest that dopamine oxidation may extend beyond general oxidative injury by enhancing iron-dependent membrane damage [16]. In this context, hydrogen peroxide generated during dopamine metabolism can undergo Fenton-type reactions in the presence of ferrous iron, leading to the formation of highly reactive hydroxyl radicals that intensify lipid oxidative damage [15,16]. At the same time, impaired GPX4-dependent detoxification of phospholipid hydroperoxides may increase membrane susceptibility to iron-driven peroxidative injury [16,25,40]. Mitochondrial dysfunction may further increase ferroptotic vulnerability by augmenting mitochondrial ROS production and disturbing intracellular iron homeostasis, thereby expanding the redox-active iron pool and promoting phospholipid peroxidation, rather than through simple iron deposition within lipid membranes [40]. Within this framework, iron does not act as an isolated trigger, but as an amplifying factor that may facilitate the transition from generalized oxidative stress to ferroptosis-related cellular vulnerability. Taken together, these observations suggest that ferroptosis should be regarded in the present review as a plausible downstream extension of dopamine-derived oxidative stress rather than as an established core mechanism in ADHD. Figure 2 illustrates the molecular cascade linking dopamine oxidation to mitochondrial dysfunction and ferroptotic pathways.

Overall, dopamine-derived oxidative stress exerts a multifaceted impact on mitochondrial function, affecting energy production, redox balance, and cell survival. These effects are particularly pronounced in dopaminergic neurons, which rely heavily on mitochondrial activity and are therefore highly sensitive to disruptions in mitochondrial homeostasis. In the context of ADHD, this mechanism may be especially relevant in fronto-striatal and mesolimbic circuits, where high energetic demands mean that even moderate impairments in mitochondrial support could contribute to inefficient signaling and reduced network stability.

## 5. Selective Vulnerability of Dopaminergic Neural Circuits in ADHD

### 5.1. Prefrontal Cortex Dysfunction and Redox-Sensitive Dopaminergic Signaling

The prefrontal cortex, particularly the dorsolateral, inferior frontal, and medial prefrontal regions, is a central node in ADHD pathophysiology because it supports inhibitory control, sustained attention, working memory, and executive regulation [41]. From a dopaminergic perspective, this region is distinctive because dopamine signaling operates within a narrow optimal range, and deviations in either direction may impair cortical efficiency [41]. This is especially relevant to a dopamine-derived oxidative stress framework, since prefrontal dysfunction in ADHD is unlikely to reflect only altered receptor signaling, but may also involve abnormal intracellular dopamine handling, with downstream effects on redox balance and synaptic performance [2]. Although direct in vivo assessment of dopamine oxidation in the human prefrontal cortex is not currently possible, convergent neuroimaging findings indicate abnormal prefrontal activity and altered frontal coupling with subcortical dopaminergic regions in ADHD, providing an anatomical context in which dopamine-related oxidative mechanisms may plausibly operate [42].

Large-scale imaging studies further support this fronto-subcortical model. In a voxel-wise mega-analysis across six cohorts, Norman et al. reported increased connectivity between striatal seeds and fronto-insular, temporal, and supplementary motor areas in 1696 youths with ADHD compared with 6737 controls, with the caudate showing the strongest effects. Importantly, altered subcortico-cortical connectivity also scaled with ADHD trait burden, suggesting that frontal dysconnectivity is dimensionally related to symptom severity rather than being limited to categorical diagnosis [43]. From a redox-centered perspective, such persistent fronto-striatal dysregulation may alter dopamine turnover in circuits that are already metabolically demanding.

Task-based evidence also points to a specifically prefrontal deficit. Salavert et al. found deficient deactivation of the medial prefrontal cortex during an N-back task in adults with ADHD, indicating impaired suppression of default mode activity during executive processing [44]. More recently, Nugiel et al. showed that methylphenidate stabilized dynamic brain network organization in stimulant-naive children with ADHD during attention and reward tasks, suggesting that abnormal prefrontal network behavior is at least partly dopamine-dependent [45]. Together, these findings support the view that cortical inefficiency in ADHD may increase compensatory dopaminergic drive, thereby enhancing intracellular dopamine exposure and vulnerability to redox imbalance.

### 5.2. Striatal Circuitry and Dopamine Turnover-Dependent Oxidative Burden

The striatum is central to any dopamine-derived oxidative stress model because it combines dense dopaminergic innervation, high dopamine turnover, substantial transporter expression, and strong relevance to ADHD-related behavior. The caudate and putamen are involved in action selection, motor control, attentional gating, and reinforcement learning, all of which are frequently altered in ADHD [41,46]. From a biochemical perspective, these regions are especially vulnerable because repeated dopamine cycling through release, reuptake, cytosolic re-entry, vesicular resequestration, and monoamine oxidase-mediated degradation creates multiple opportunities for quinone formation, hydrogen peroxide production, and aldehyde stress [41,46].

This vulnerability is supported by molecular imaging data. In drug-naïve adults with ADHD, Itagaki et al. reported reduced dopamine transporter availability relative to controls, together with correlations between transporter abnormalities and symptom dimensions [47]. Although DAT availability does not directly measure oxidative stress, it reflects altered presynaptic dopamine handling. Because DAT strongly influences how much extracellular dopamine re-enters the cytosol, such abnormalities have direct implications for the oxidation-prone cytosolic dopamine pool [47].

Large-scale functional imaging further highlights the caudate as a key locus. Norman et al. found that the most prominent ADHD-related signal in their mega-analysis involved caudate connectivity, with stronger coupling to cortical regions responsible for salience processing and cognitive control [43]. This is particularly relevant because the caudate is both richly dopaminergic and critically involved in executive dysfunction. A circuit that is repeatedly over-engaged or inefficiently regulated would be expected to require increased dopamine flux, thereby increasing the burden of dopamine auto-oxidation and MAO-dependent metabolism.

Longitudinal evidence also indicates that striatal circuitry in ADHD remains biologically plastic. Using ABCD data, Kaminski et al. showed that stimulant exposure over two years was associated with changes in striatal functional connectivity in childhood ADHD [48]. Although this does not directly assess oxidation, it suggests that therapies altering dopamine trafficking, storage, or signaling may also influence oxidative liability [48]. Animal work supports this developmental vulnerability. In a 2024 rat ADHD model, Bogdańska-Chomczyk et al. described postnatal alterations in striatal architecture and biochemical markers, consistent with the view that the striatum is a biologically vulnerable site in which dopamine-related metabolic stress may accumulate over development [49].

### 5.3. Mesocorticolimbic Pathway and Reward-Related Oxidative Dynamics

The mesocorticolimbic pathway, connecting the ventral tegmental area with the prefrontal cortex and nucleus accumbens, is essential for reward anticipation, motivational salience, reinforcement learning, and effort allocation, all of which are altered in ADHD [50]. This pathway is particularly relevant to the present framework because reward-related dopaminergic signaling is phasic and behavior-dependent. Repeated abnormalities in cue processing or reinforcement sensitivity may therefore produce repeated surges in dopamine turnover, increasing the likelihood of dopamine oxidation, especially when vesicular sequestration or transporter function is suboptimal.

Functional MRI studies support mesolimbic dysfunction in ADHD. Furukawa et al. showed that individuals with ADHD had reduced activation to cues predicting affiliative reward in the bilateral ventral and dorsal striatum, together with increased ventral striatal activation during reward delivery [51]. Mechanistically, this pattern suggests inefficient anticipatory coding with altered downstream reward reactivity, a configuration likely to disrupt normal dopamine timing and turnover. In a dopamine-derived ROS framework, such dysregulated phasic signaling may be more important than mean dopamine levels alone, because oxidative risk depends strongly on how frequently dopamine cycles through release, reuptake, and intracellular metabolism.

Medication studies further reinforce the reward-circuit argument. In adults with ADHD, Furukawa et al. also found that methylphenidate increased ventral striatal differentiation between reward and non-reward cues and reduced dorsal striatal–dorsomedial prefrontal coupling during reward outcome processing [52]. These findings suggest that stimulant treatment may improve reward-related signaling not simply by increasing catecholamine tone, but by normalizing circuit-level inefficiency that would otherwise sustain maladaptive dopamine cycling and oxidative burden [52].

More recently, Zaher et al. reported that nucleus accumbens functional connectivity predicted clinical course in adult ADHD, with accumbens-centered connectivity patterns differing according to medication adherence and longitudinal outcome [53]. Because the accumbens is a classic dopamine-rich reward hub, these data support the view that mesolimbic circuit organization is closely tied to disease persistence and may represent a plausible substrate for chronic dopamine-related redox stress [53].

### 5.4. Regional Differences in Antioxidant Capacity and Dopaminergic Vulnerability

A key principle of selective vulnerability is that dopamine-rich regions are not equally protected against the biochemical consequences of dopamine metabolism. Vulnerability depends on the local balance among dopamine synthesis, reuptake, vesicular sequestration, mitochondrial load, metal availability, and antioxidant buffering [54]. Thus, the same neurotransmitter can be relatively well tolerated in one region and pro-oxidative in another, depending on its compartmentalization efficiency and detoxification capacity.

The striatum is particularly important in this regard because of its high DAT expression and intense dopamine recycling. The prefrontal cortex, in contrast, has lower DAT density and relies more heavily on enzymatic clearance and broader catecholamine regulation. These regional differences imply that oxidative pressure may emerge through partially different mechanisms across circuits. In the striatum, the main risk may arise from rapid recapture and repeated cytosolic exposure, whereas in the prefrontal cortex the risk may be linked more to inefficient modulation, prolonged extracellular signaling, and repeated compensatory catecholamine demand. The clinical imaging literature in ADHD is consistent with this division, repeatedly implicating both fronto-cortical dysfunction and striatal dysconnectivity rather than a single isolated node [43].

Recent non-human work also suggests that developmental and circuit-specific dopaminergic differences matter. Aydin and Adiguzel examined the mesocortical dopaminergic system in spontaneously hypertensive rats, a classic ADHD model, and concluded that mesocortical anatomy alone could not explain hyperactivity. Although negative in one sense, the study is still informative because it argues against overly simplistic one-region explanations and supports a distributed-circuit model, which is exactly the framework in which oxidative burden would be expected to vary across anatomically and functionally distinct dopamine systems [55].

Taken together, the available evidence supports the view that ADHD involves a distributed disturbance of dopamine-relevant circuitry, with the prefrontal cortex, caudate, broader striatum, and nucleus accumbens representing particularly important sites of dysfunction. Within the conceptual boundaries of this review, these circuits are not only functionally abnormal but also biochemically plausible sites of dopamine-derived oxidative stress, because each combines altered dopamine handling with high metabolic demand and region-specific limitations in redox buffering [43].

## 6. Dopamine-Derived Oxidative Stress in ADHD: Evidence Base

### 6.1. Peripheral Biomarkers Reflecting Dopamine-Related Oxidative Imbalance

Clinical evidence supporting oxidative stress involvement in ADHD has increasingly emerged from studies evaluating peripheral redox biomarkers. Although these markers are indirect and do not isolate dopamine-derived ROS specifically, several studies provide consistent signals of systemic oxidative imbalance that are mechanistically compatible with dopaminergic dysregulation. It should be emphasized that these biomarkers were assessed in peripheral biological matrices, including blood-derived samples, plasma, and urine, depending on the study design. Therefore, they reflect systemic oxidative or nitrosative status rather than direct central nervous system dopamine oxidation. Their interpretation in relation to dopamine-derived oxidative stress in ADHD must consequently remain indirect and inferential.

A recent clinical study published in 2024 evaluated oxidative and nitrosative stress markers in blood samples from 59 ADHD patients before and after methylphenidate treatment. The authors reported measurable alterations in oxidative status and antioxidant defense systems, indicating that ADHD is associated with a disturbed redox balance and that pharmacological modulation of dopamine transmission influences this equilibrium [56]. This is particularly relevant because methylphenidate directly alters dopamine availability, suggesting that at least part of the oxidative signature may be linked to dopamine handling rather than being purely systemic.

More recent data further support this link. In a 2026 randomized controlled trial analyzing children with ADHD, plasma-based oxidative stress parameters such as glutathione peroxidase activity, glutathione reductase, and reactive oxygen metabolites were significantly associated with clinical outcomes and nutritional status [57]. Importantly, these antioxidant systems are directly involved in detoxifying hydrogen peroxide and lipid peroxides, which are key downstream products of dopamine oxidation.

Complementary evidence comes from Mendelian randomization studies exploring causal relationships between circulating oxidative biomarkers and psychiatric disorders. One such study reported that ADHD is associated with decreased levels of key antioxidant molecules, including ascorbate and catalase, suggesting a systemic reduction in antioxidant buffering capacity [58]. Although these findings do not isolate dopamine-specific ROS, they support the presence of a redox imbalance that would amplify the effects of dopamine oxidation in neuronal environments.

Taken together, peripheral biomarker studies indicate that ADHD is associated with measurable oxidative stress and impaired antioxidant defenses, providing a systemic context in which dopamine-derived oxidative mechanisms may exert stronger biological effects.

### 6.2. Genetic Susceptibility Affecting Dopamine Oxidation Pathways

Genetic studies in ADHD have consistently implicated components of the dopaminergic system, many of which are directly relevant to the regulation of intracellular dopamine levels and, by extension, its oxidative fate. Variants in genes such as DAT1 (SLC6A3), COMT, and monoamine oxidases influence dopamine turnover, synaptic availability, and intracellular metabolism, all of which determine the size of the cytosolic dopamine pool [59].

While classical ADHD genetics has focused on neurotransmission, emerging work suggests that these same genetic factors may indirectly modulate oxidative stress. For example, alterations in DAT function influence the rate of dopamine reuptake and cytosolic re-entry, thereby affecting exposure to oxidation pathways [60]. Similarly, COMT polymorphisms alter dopamine degradation dynamics, particularly in the prefrontal cortex, potentially modifying local oxidative burden [61].

Recent experimental evidence reinforces the interaction between dopamine transport and oxidative stress. A 2025 study using an ADHD animal model demonstrated that high oxidative stress disrupts the balance between DAT and VMAT2 function, leading to increased cytosolic dopamine and impaired vesicular storage [20]. This finding is particularly important because it links oxidative stress directly to dopamine compartmentalization, creating a bidirectional relationship in which dopamine dysregulation promotes oxidative stress, which in turn further disrupts dopamine handling.

Additionally, metabolomic approaches are beginning to reveal biochemical signatures associated with ADHD that include pathways related to dopamine metabolism and redox balance. Although still emerging, these studies suggest that genetic susceptibility and metabolic phenotype converge on pathways that regulate both dopamine turnover and oxidative stress [62].

### 6.3. Experimental Models Linking Dopamine Turnover to Oxidative Stress

Animal models have provided some of the most direct evidence linking dopamine dysregulation to oxidative stress in ADHD-relevant contexts. The spontaneously hypertensive rat (SHR), one of the most widely used ADHD models, has been shown to exhibit both dopaminergic abnormalities and increased oxidative stress markers.

In the 2025 study mentioned above, SHR animals displayed elevated levels of oxidative damage markers such as 8-hydroxy-2′-deoxyguanosine, along with reduced antioxidant capacity, indicating DNA oxidation and impaired redox homeostasis [20]. These changes were accompanied by alterations in dopamine transporter systems, suggesting that oxidative stress and dopamine dysregulation are tightly coupled processes rather than independent phenomena.

Importantly, the same study demonstrated that activation of the Nrf2/Keap1/HO-1 antioxidant pathway restored dopamine transport balance, normalizing both DAT and VMAT2 function. This provides mechanistic evidence that oxidative stress is not merely a consequence of dopamine dysfunction but can actively drive abnormalities in dopamine handling, reinforcing a feedback loop between redox imbalance and neurotransmission.

Other experimental work has shown that oxidative stress can alter synaptic plasticity, mitochondrial function, and neuronal excitability in dopaminergic circuits [63,64,65], further supporting the biological plausibility of dopamine-derived oxidative mechanisms in ADHD-like phenotypes.

### 6.4. Indirect Neuroimaging Correlates of Dopamine Turnover and Oxidative Vulnerability

Although current neuroimaging techniques cannot directly measure dopamine-derived ROS in vivo, imaging studies provide indirect evidence of altered dopamine turnover and circuit dysfunction in ADHD. These findings are relevant because dopamine oxidation is strongly dependent on turnover dynamics rather than static dopamine levels.

PET imaging studies have consistently reported alterations in dopamine transporter availability and receptor binding in ADHD, indicating dysregulated dopamine signaling [66]. Changes in DAT availability, in particular, are highly relevant because they influence the rate of dopamine reuptake and cytosolic exposure, which are key determinants of oxidative risk [67].

Functional MRI studies further demonstrate abnormalities in fronto-striatal and mesocorticolimbic circuits, which are heavily dopaminergic and metabolically active [68]. Large-scale analyses have shown altered connectivity patterns involving the caudate, prefrontal cortex, and reward-related regions, suggesting persistent dysregulation of dopamine-dependent signaling networks [69,70,71].

From a mechanistic perspective, these imaging findings can be interpreted as reflecting circuits with altered dopamine dynamics, including abnormal release, reuptake, and intracellular processing. Such conditions are expected to increase the probability of dopamine oxidation, particularly in regions with high metabolic demand and limited antioxidant capacity.

Thus, while neuroimaging does not directly measure oxidative stress, it provides critical contextual evidence linking dopamine dysregulation to the anatomical and functional substrates where dopamine-derived ROS are most likely to exert their effects.

Taken together, the available evidence supports the biological plausibility of this framework, while also highlighting important limitations in ADHD-specific mechanistic data. These evidence domains are summarized in Supplementary Table S1.

## 7. Neurodevelopmental Impact of Dopamine-Derived Oxidative Stress

### 7.1. Oxidative Modulation of Synaptic Pruning

Synaptic pruning is a core neurodevelopmental process through which immature neural circuits are refined during childhood and adolescence. In ADHD, this developmental period overlaps with the emergence and consolidation of attentional, executive, and behavioral symptoms, making pruning-related mechanisms highly relevant. However, direct evidence that dopamine-derived oxidative stress alters synaptic pruning specifically in ADHD remains limited. At present, the strongest support is indirect and rests on two converging lines of evidence: first, ADHD is a neurodevelopmental disorder with reproducible abnormalities in fronto-subcortical maturation and connectivity [72], and second, oxidative stress is capable of perturbing pruning-related developmental pathways in other neurodevelopmental contexts [73].

The most defensible interpretation, therefore, is that dopamine-derived ROS may act as a modulatory factor during pruning rather than as a fully established primary driver in ADHD. This inference is biologically plausible because dopamine-rich circuits, especially fronto-striatal pathways, are under heavy developmental remodeling and are also regions in which altered dopamine handling is repeatedly implicated in ADHD. Norman et al. showed in a large voxel-wise mega-analysis that ADHD is associated with subcortico-cortical dysconnectivity, especially involving the caudate and distributed cortical regions, supporting the idea that atypical circuit refinement is part of the disorder’s developmental architecture [43]. In parallel, Joseph et al. reported in a meta-analysis that ADHD is associated with evidence of oxidative stress, even though antioxidant findings were less consistent, suggesting a redox environment that could amplify developmentally relevant synaptic vulnerability [74].

Because direct ADHD-specific pruning studies tied to dopamine oxidation are lacking, any stronger claim would overstate the evidence. A careful review should therefore state that dopamine-derived oxidative stress is a candidate developmental modulator of pruning in ADHD, supported by neurodevelopmental timing, circuit localization, and oxidative biology, but not yet proven by direct mechanistic human data [75].

### 7.2. Effects on Dendritic Spine Maturation

The evidence is substantially stronger for dendritic spine maturation than for pruning. Dendritic spines are dynamic structures that reflect the maturation and stabilization of excitatory synapses, and altered spine morphology is directly relevant to ADHD because it affects network efficiency, signal integration, and plasticity. In a prenatal nicotine exposure mouse model of ADHD, Contreras et al. showed reduced dendritic spine density in hippocampal pyramidal neurons, together with a shift toward a higher proportion of immature thin spines and a lower proportion of mature mushroom spines [76]. Importantly, methylphenidate restored both behavioral abnormalities and the structural spine phenotype, linking ADHD-like behavior, neuroplasticity deficits, and abnormal spine maturation in the same experimental system.

Although this study did not directly quantify dopamine-derived quinones or cytosolic dopamine oxidation, it provides evidence that an ADHD-like developmental phenotype includes impaired synaptic maturation at the level of dendritic spine structure and that catecholaminergic modulation can partially normalize this phenotype [76]. In the context of the present review, we interpret these findings as compatible with the hypothesis that abnormal dopamine handling during development may influence structural synaptic maturation through mechanisms that could include redox-sensitive signaling and protein modification pathways discussed in earlier sections.

Additional, albeit less ADHD-specific, support comes from developmental stimulant studies showing that methylphenidate can reshape dendritic spine organization and dendritic complexity in cortical and striatal regions. For example, Adriani et al. found that methylphenidate treatment recovered stress-induced abnormalities in dendritic spine density in rodent dorsal anterior cingulate cortex, indicating that developing cortical connectivity remains structurally plastic and catecholamine-sensitive [77]. Although this model is not an ADHD model per se, it reinforces the broader conclusion that developmental catecholaminergic perturbation is tightly coupled to spine remodeling in brain regions relevant to executive control.

### 7.3. Dopamine–ROS Interaction with Neuroplasticity

Neuroplasticity is one of the most plausible developmental domains through which dopamine-derived oxidative stress may influence ADHD trajectories. Dopamine is a major modulator of long-term potentiation, AMPA receptor trafficking, synaptic gain, and experience-dependent adaptation, while reactive oxygen species can act either as physiological signaling molecules or as pathological disruptors depending on concentration, compartment, and duration. In ADHD-relevant models, the most informative original study remains the work by Contreras et al., who demonstrated reduced hippocampal LTP, altered AMPA receptor subunit composition and distribution, and impaired dendritic spine maturation in the prenatal nicotine exposure model, all of which were restored by methylphenidate [76]. This is important because it ties behavioral impairment to measurable defects in synaptic plasticity rather than treating neuroplasticity as a vague downstream concept.

The relevance to dopamine-derived oxidative stress lies in the fact that plasticity-related signaling is highly redox-sensitive. When dopamine metabolism shifts toward greater cytosolic accumulation and oxidation, the resulting ROS and quinone species may contribute to altered receptor trafficking, synaptic protein modification, and impaired mitochondrial support for plasticity. While direct demonstrations of this exact sequence in ADHD tissue are still unavailable, the combination of altered catecholaminergic signaling, measurable oxidative imbalance, and plasticity deficits in ADHD models makes this mechanism biologically credible. Clinical support for altered systemic redox state in ADHD also comes from more recent work showing changes in peripheral blood-derived oxidative and nitrosative stress markers before and after methylphenidate treatment, as well as plasma and urine-based markers of nitric oxide metabolism and oxidative stress, implying that pharmacological dopamine modulation is accompanied by measurable shifts in peripheral redox biology [56,78].

A cautious but academically defensible conclusion is that dopamine-derived ROS do not replace classical neurotransmitter-based models of ADHD neuroplasticity, but may help explain how those systems shift toward maladaptive plastic states, especially during developmental windows when synapses are being stabilized, remodeled, or eliminated.

### 7.4. Long-Term Circuit Remodeling and Developmental Trajectory in ADHD

The long-term developmental impact of dopamine-derived oxidative stress is most plausibly expressed at the level of circuit remodeling. ADHD is increasingly understood as a disorder of altered developmental trajectories rather than a static lesion model, and large imaging studies support this view. Norman et al. demonstrated robust subcortico-cortical dysconnectivity in ADHD across multiple cohorts, particularly involving the caudate and widespread cortical targets, suggesting that developmental circuit organization is persistently altered [43]. More recent structural work has also reported distinctive cortical morphology in young adults diagnosed with and medicated for ADHD, reinforcing the idea that ADHD is associated with durable anatomical variation rather than only transient childhood dysfunction [79,80].

Animal models also support the developmental relevance of this interpretation. In juvenile spontaneously hypertensive rats, Kozłowska et al. found increased oxidative stress markers together with reduced medial prefrontal cortex volume and altered dopaminergic features, including changes related to dopamine D2 receptor signaling in the same region [81]. Notably, some of these abnormalities shifted with maturation, suggesting that oxidative and dopaminergic disturbances may be especially important during early developmental stages rather than remaining identical across the lifespan. This age-dependent pattern is highly relevant to ADHD because it fits a model in which dopamine-related redox stress exerts its greatest impact during periods of active cortical and subcortical remodeling.

Additional support comes from developmental rodent models in which early-life perturbations produce both ADHD-like behavior and dopaminergic circuit dysfunction. Bock et al. showed that early life stress induced ADHD-like behavioral changes together with metabolic hypoactivity in prefrontal, mesolimbic, and subcortical regions, and that methylphenidate normalized both behavior and brain metabolic dysfunction [82]. Although oxidative stress was not directly quantified in this study, it reinforces the broader developmental point that catecholamine-sensitive circuits in ADHD-like states undergo persistent remodeling that remains pharmacologically modifiable.

Overall, the available evidence supports a restrained but coherent conceptual model in which dopamine-derived oxidative stress may contribute to long-term circuit remodeling in ADHD by interacting with developmental processes such as synaptic plasticity, spine maturation, and region-specific maturation of fronto-striatal and mesolimbic networks.

## 8. Therapeutic Implications Focused on Dopamine-Related Redox Mechanisms

### 8.1. Impact of Psychostimulants on Dopamine Turnover and Oxidative Stress

Psychostimulants, particularly methylphenidate and amphetamine derivatives, constitute the first-line pharmacological treatment for ADHD and exert their therapeutic effects mainly through modulation of dopaminergic and noradrenergic neurotransmission. At the presynaptic level, methylphenidate inhibits the dopamine transporter, thereby increasing extracellular dopamine availability, whereas amphetamines additionally stimulate reverse transport and vesicular release. From a redox perspective, this raises an apparent paradox: while increased dopamine availability might theoretically favor greater cytosolic dopamine exposure and oxidation, psychostimulants nonetheless produce clear therapeutic benefits in clinical practice [83].

Experimental data suggest that the net effect of psychostimulants is context-dependent and may differ between acute and chronic exposure. In an animal study, chronic methylphenidate administration was associated with increased oxidative stress markers in specific brain regions, including elevated lipid peroxidation and altered antioxidant enzyme activity, suggesting that excessive or prolonged dopaminergic stimulation may have pro-oxidative effects [84]. However, these findings must be interpreted cautiously, as they often involve supratherapeutic dosing or non-clinical conditions.

Conversely, clinical data indicate that methylphenidate may normalize peripheral oxidative imbalance in ADHD patients. In a 2024 study, treatment with methylphenidate was associated with modulation of blood-derived oxidative and nitrosative stress markers, suggesting a potential stabilizing effect on systemic redox homeostasis [56]. This apparent contradiction can be reconciled by considering that improved dopaminergic signaling efficiency reduces maladaptive dopamine turnover and circuit-level inefficiency, thereby potentially decreasing the pathological fraction of cytosolic dopamine prone to oxidation.

Recent systems neuroscience studies further support this interpretation. Methylphenidate has been shown to stabilize dynamic brain network organization in ADHD, particularly in circuits involved in attention and reward processing, suggesting that therapeutic benefit may arise from restoring efficient dopaminergic signaling rather than simply increasing dopamine levels [45]. Within this framework, optimized dopamine signaling may reduce the need for compensatory hyperactivation and excessive turnover, indirectly limiting dopamine-derived oxidative stress.

Importantly, psychostimulants are clinically established in ADHD, whereas their interpretation within a dopamine-derived oxidative stress framework remains mechanistic and inferential rather than directly demonstrated.

### 8.2. Targeting Cytosolic Dopamine Accumulation and Vesicular Dysfunction

Given that cytosolic dopamine represents the primary substrate for oxidative reactions, strategies aimed at enhancing vesicular sequestration or reducing cytosolic accumulation are of particular interest. The vesicular monoamine transporter VMAT2 plays a central role in this process, and its function directly determines the balance between protected vesicular dopamine and oxidation-prone cytosolic dopamine [85].

Experimental studies have demonstrated that impaired VMAT2 function leads to increased cytosolic dopamine and enhanced oxidative stress, whereas restoration of vesicular storage capacity reduces oxidative damage and neuronal vulnerability. In a recent study using human dopaminergic neurons derived from induced pluripotent stem cells, VMAT2 dysfunction was shown to promote dopamine oxidation and neurodegeneration, highlighting vesicular transport as a key upstream determinant of redox homeostasis [14].

Although direct pharmacological targeting of VMAT2 in ADHD is not currently part of clinical practice, this pathway represents a conceptual therapeutic target. Interventions that improve vesicular storage efficiency or reduce cytosolic dopamine exposure could theoretically limit dopamine-derived ROS generation without suppressing physiological neurotransmission.

In addition, modulation of MAO activity may influence the balance between dopamine metabolism and oxidative stress. However, because MAO inhibition increases dopamine availability, its net effect on oxidative stress is complex and may depend on the relative contributions of enzymatic versus non-enzymatic oxidation pathways [86].

At present, strategies aimed at reducing cytosolic dopamine accumulation or improving vesicular sequestration should be regarded as conceptual or preclinical in ADHD. Their relevance lies primarily in clarifying potentially actionable upstream mechanisms rather than in supporting an immediately translatable treatment approach.

### 8.3. Antioxidant Strategies Targeting Dopamine Oxidation Pathways

Antioxidant therapies represent a complementary approach aimed at mitigating the downstream effects of dopamine-derived oxidative stress. Among these, *N*-acetylcysteine (NAC) has attracted particular attention due to its role as a precursor of glutathione, the major intracellular antioxidant. NAC has been shown to restore glutathione levels, reduce oxidative stress, and modulate glutamatergic and dopaminergic signaling [87,88].

Clinical studies in neuropsychiatric disorders suggest that NAC may improve symptoms associated with impulsivity and cognitive dysfunction, although data specifically in ADHD remain limited. Nevertheless, its mechanistic relevance is strong, given its capacity to neutralize quinone intermediates and enhance detoxification pathways [89].

Other micronutrients, including zinc and selenium, have also been investigated in ADHD. Zinc is known to modulate dopamine transporter function and synaptic signaling, while selenium is a cofactor for antioxidant enzymes such as glutathione peroxidase. Meta-analyses have reported that zinc supplementation may improve ADHD symptoms, particularly in individuals with baseline deficiency, suggesting that restoring micronutrient-dependent antioxidant capacity may have therapeutic benefits [90,91].

Emerging experimental approaches also include quinone scavengers and metal chelators, which directly target key steps in dopamine oxidation. For example, glutathione and cysteine can form conjugates with dopamine quinones, preventing protein modification and interrupting redox cycling. Similarly, iron chelators can reduce Fenton chemistry and limit the formation of highly reactive hydroxyl radicals [92].

Overall, antioxidant interventions in ADHD remain adjunctive and incompletely validated. While NAC and selected micronutrients may have preliminary translational relevance, more targeted strategies such as quinone scavenging or iron-modulating approaches remain highly exploratory in ADHD.

### 8.4. Precision Medicine Approaches and Future Therapeutic Directions

The heterogeneity of ADHD suggests that a one-size-fits-all approach to treatment may be insufficient. From a dopamine-derived oxidative stress perspective, individual differences in dopamine metabolism, antioxidant capacity, and genetic background may determine susceptibility to oxidative damage and response to therapy [93].

Recent advances in biomarker research and systems biology offer the possibility of identifying subgroups of patients characterized by distinct redox and dopaminergic profiles. For example, patients with reduced antioxidant capacity or altered dopamine transporter function may benefit from targeted antioxidant or dopamine-modulating therapies [94,95,96].

The integration of multi-omics approaches, including genomics, metabolomics, and proteomics, may further refine this stratification. In addition, artificial intelligence-based models could be used to integrate complex datasets and predict individual treatment responses based on redox and dopaminergic parameters [97,98].

Ultimately, therapeutic strategies combining optimization of dopaminergic signaling with targeted modulation of oxidative stress may represent a promising and mechanistically grounded direction for future ADHD research and treatment development. These precision-oriented strategies should currently be considered forward-looking and hypothesis-generating rather than ready for routine clinical implementation in ADHD. Their value lies in framing future stratified research, particularly in identifying subgroups in whom dopaminergic and redox-related mechanisms may be more biologically relevant.

## 9. Conceptual Model of Dopamine-Derived Oxidative Stress in ADHD

**Figure 3:**
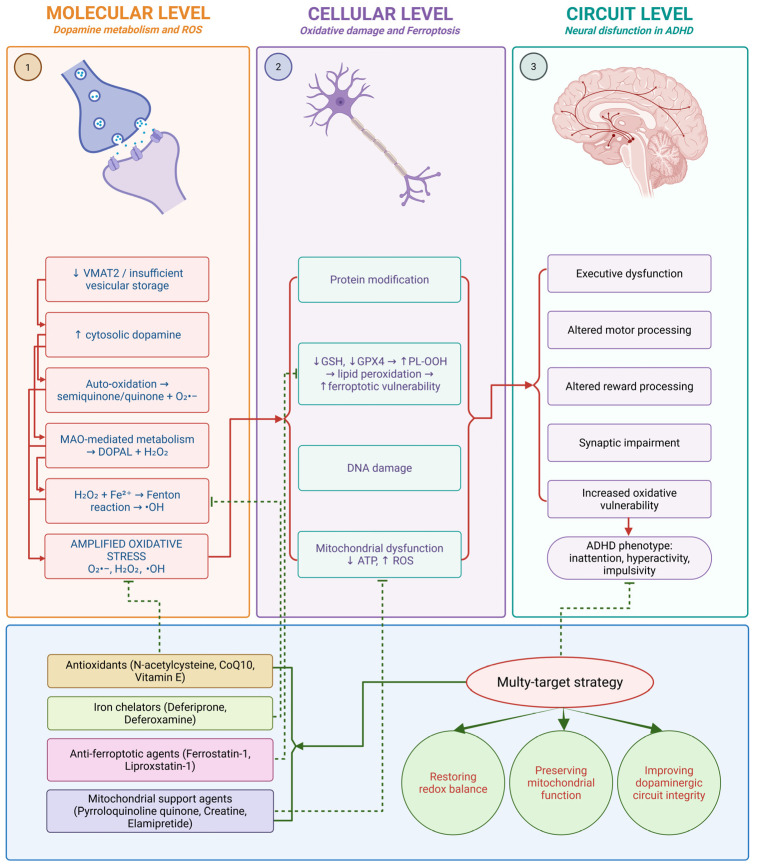
Integrated model linking dopamine-derived oxidative stress to molecular, cellular, and circuit-level dysfunction in ADHD and associated therapeutic targets. At the molecular level, reduced VMAT2-mediated vesicular storage increases cytosolic dopamine availability, favoring dopamine auto-oxidation and MAO-mediated metabolism. These pathways generate reactive intermediates, including semiquinone/quinone species and DOPAL, together with reactive oxygen species (ROS), such as O_2_^•−^ and H_2_O_2_. In the presence of Fe^2+^, H_2_O_2_ undergoes Fenton reactions, leading to ^•^OH formation and amplification of oxidative stress. At the cellular level, dopamine-derived oxidative stress promotes protein modification, DNA damage, mitochondrial dysfunction, and lipid peroxidation. In parallel, decreased glutathione (GSH) and glutathione peroxidase 4 (GPX4) activity impair phospholipid hydroperoxide detoxification, resulting in increased PL-OOH accumulation, enhanced lipid peroxidation, and increased ferroptotic vulnerability. At the circuit level, these changes may contribute to executive dysfunction, altered motor and reward processing, synaptic impairment, and increased oxidative vulnerability, ultimately converging into the ADHD phenotype characterized by inattention, hyperactivity, and impulsivity. The lower panel summarizes potential therapeutic strategies, including antioxidants, iron chelators, anti-ferroptotic agents, and mitochondrial support agents, aimed at restoring redox balance, preserving mitochondrial function, and improving dopaminergic circuit integrity. Graphical notation: red lines indicate proposed pathophysiological relationships within the dopamine-derived oxidative stress cascade, whereas green lines indicate proposed therapeutic or modulatory actions. Solid lines represent mechanistic or downstream relationships within the model, while dashed lines represent hypothesized modulatory or therapeutic effects. Arrowheads indicate promoting, downstream, or contributory effects, whereas T-bars indicate inhibitory, reducing, or attenuating effects on the targeted process, including the attenuation of downstream cellular dysfunction and ADHD-related phenotypic expression. Created in BioRender. Ciobarceanu, G. (2026) https://BioRender.com/tl3jsjf (accessed on 7 May 2026).

The integration of biochemical, neurodevelopmental, and circuit-level evidence allows the formulation of a coherent mechanistic model in which dopamine-derived oxidative stress may represent an intermediate linking dopaminergic dysregulation to neural circuit dysfunction in ADHD. Rather than viewing dopamine abnormalities solely in terms of synaptic signaling deficits, this model emphasizes intracellular dopamine handling and its redox consequences as a potentially important, yet underexplored, dimension of ADHD pathophysiology. This integrated multi-level model is illustrated in Figure 3.

At the core of this framework is the concept that the cytosolic fraction of dopamine, rather than total dopamine levels, represents the primary determinant of oxidative risk. Experimental evidence has demonstrated that increases in cytosolic dopamine, whether through enhanced synthesis, impaired vesicular sequestration, or altered transporter dynamics, lead to elevated production of reactive oxygen species and reactive dopamine metabolites [85,99]. Mosharov et al. directly measured cytosolic dopamine in dopaminergic neurons and showed that increases in intracellular dopamine correlate with enhanced oxidative stress and neuronal vulnerability, providing a foundational experimental basis for this model [18].

In the context of ADHD, alterations in dopamine transporter function and synaptic regulation have been consistently reported. Imaging studies have demonstrated abnormal dopamine transporter availability and dysregulated fronto-striatal connectivity, indicating that dopamine handling is fundamentally altered in key neural circuits [47,100]. Norman et al. further showed that ADHD is associated with widespread subcortico-cortical dysconnectivity, particularly involving the caudate and prefrontal cortex, suggesting that dopaminergic circuit inefficiency is a central feature of the disorder [43].

Within this altered circuit environment, inefficient dopaminergic signaling may lead to compensatory increases in dopamine turnover. Increased release, reuptake, and metabolism of dopamine may elevate the probability of cytosolic accumulation, particularly when vesicular storage mechanisms are insufficient or overwhelmed. In turn, such conditions may favor dopamine auto-oxidation and MAO-mediated metabolism, with subsequent generation of H_2_O_2_, quinones, and reactive aldehydes such as DOPAL.

Recent experimental work has further demonstrated that dopamine oxidation is not merely a byproduct of dysregulated neurotransmission but can actively drive cellular dysfunction. Sun et al. showed that dopamine oxidation promotes degradation of glutathione peroxidase 4, linking dopamine metabolism directly to impaired antioxidant defense and ferroptotic cell death pathways. This finding provides mechanistic evidence that dopamine-derived oxidative stress can propagate beyond initial redox imbalance to affect broader cellular survival systems [16].

At the circuit level, regions most consistently implicated in ADHD, including the prefrontal cortex, striatum, and mesolimbic pathways [101,102], appear to share several features that may increase susceptibility to dopamine-derived oxidative stress. These include high dopamine turnover, significant metabolic demand, and region-specific limitations in antioxidant capacity. Functional imaging studies have consistently demonstrated abnormalities in these circuits, including altered connectivity and inefficient activation patterns, suggesting that these regions operate under persistent physiological strain [42,43,44,45,50,51,52,53,68,69,70,101].

This model proposes a potential feed-forward mechanism in which dopaminergic dysregulation may increase cytosolic dopamine exposure, with subsequent enhancement of oxidative stress that may impair synaptic function, mitochondrial integrity, and neuronal signaling. These alterations may, in turn, exacerbate circuit inefficiency, increase the demand for dopaminergic compensation, and help perpetuate the cycle. Over time, this process may contribute to the neurodevelopmental alterations observed in ADHD, including delayed cortical maturation and persistent network dysfunction.

Importantly, this conceptual framework does not replace existing neurotransmitter-based models of ADHD but rather extends them by incorporating intracellular and redox dimensions of dopamine biology. It provides a plausible mechanistic bridge between molecular processes, such as dopamine oxidation and protein modification, and higher-level phenomena, including circuit dysfunction and behavioral symptoms [47].

Moreover, this model has potential translational implications. By highlighting cytosolic dopamine and its oxidative derivatives as potentially important pathogenic factors, this framework suggests that therapeutic strategies may need to consider not only synaptic dopamine levels but also intracellular dopamine handling and oxidative balance. This perspective may help explain variability in treatment response and supports the exploration of adjunctive therapies targeting redox mechanisms.

## 10. Limitations and Future Directions

The specific contribution of dopamine-derived oxidative stress to ADHD remains incompletely defined. A major limitation of the current evidence base is the absence of direct in vivo biomarkers capable of measuring dopamine oxidation in the living human brain. As a result, much of the available literature remains indirect, drawing on peripheral oxidative markers, neuroimaging correlates, genetic susceptibility data, and experimental models rather than direct confirmation of dopamine-specific oxidative injury in patients with ADHD.

Additional limitations arise from the marked heterogeneity of ADHD phenotypes, which may obscure biologically meaningful subgroup differences in dopaminergic and redox-related mechanisms. Interpretation is further complicated by medication status, since psychostimulant exposure may influence both dopamine handling and oxidative parameters, making it difficult to distinguish disease-related findings from treatment-related effects.

Another important limitation is the uncertain correspondence between peripheral oxidative biomarkers and central nervous system redox processes. Although peripheral markers provide useful systemic information, they cannot reliably establish the presence, magnitude, or regional specificity of dopamine-derived oxidative stress within the brain. In parallel, a substantial part of the mechanistic framework discussed in this review is extrapolated from broader dopaminergic research, including Parkinsonian and other non-ADHD experimental models, which may not fully reflect the developmental and functional characteristics of ADHD.

Future research should therefore focus on longitudinal, multimodal, and subgroup-oriented approaches capable of integrating peripheral biomarkers, neuroimaging, metabolomics, and functional assessments of dopaminergic circuits. Greater priority should be given to identifying biologically meaningful ADHD subgroups and to developing more specific translational markers of dopamine oxidation and redox imbalance in vivo. Overall, further validation is required before dopamine-derived oxidative stress can be considered a clinically actionable framework in ADHD.

## 11. Conclusions

Dopamine-derived oxidative stress may represent a biologically plausible extension of current neurobiological models of ADHD by linking dopaminergic dysregulation to intracellular redox imbalance. Because dopamine is a redox-active molecule, altered dopamine handling may increase oxidative vulnerability in frontostriatal and mesocorticolimbic circuits relevant to ADHD, particularly under conditions of increased cytosolic accumulation.

Although direct evidence for dopamine-specific oxidative mechanisms in ADHD remains limited, the available molecular, experimental, and systems-level findings support the biological plausibility of this framework. At present, dopamine-derived oxidative stress should be viewed not as an established unifying mechanism in ADHD, but as a biologically plausible and testable framework that may help connect dopaminergic dysfunction with oxidative imbalance, circuit dysfunction, and neurodevelopmental vulnerability. Its main value lies in guiding future biomarker-based, experimental, and translational research aimed at clarifying whether redox-related processes define biologically meaningful ADHD subgroups.

## Data Availability

No new data were created or analyzed in this study. Data sharing is not applicable to this article.

## Supplementary Materials

The following supporting information can be downloaded at: https://www.mdpi.com/article/10.3390/antiox15050613/s1, Table S1. Summary of evidence supporting the dopamine-derived oxidative stress framework in attention-deficit/hyperactivity disorder.
