# Supplementary material for: Dopamine-Derived Oxidative Stress in Attention-Deficit/Hyperactivity Disorder: A Narrative Review of Molecular Mechanisms, Neural Circuitry, and Therapeutic Implications
Source: Antioxidants (Basel). 2026 May 13;15(5):613. doi: 10.3390/antiox15050613 (PMC13203595; doi:10.3390/antiox15050613)
Supplement: Supplementary file 1 [file antioxidants-15-00613-s001.zip › antioxidants-4276430-supplementary.pdf]

Table S1. Summary of evidence supporting the dopamine-derived oxidative stress framework in attention-deficit/hyperactivity disorder

Domain	Summary of evidence	Key references	Type of evidence	ADHD specificity	Main limitation
**Peripheral oxidative biomarkers**	Clinical studies in ADHD have reported alterations in oxidative and antioxidant parameters, including changes in glutathione-related enzymes, reactive oxygen metabolites, and nitrosative stress markers. Some studies also suggest partial modulation of these markers after methylphenidate treatment.	[56,58,74]	Human clinical and observational studies	ADHD-specific, but indirect	Peripheral biomarkers do not directly demonstrate dopamine-derived oxidative stress in the central nervous system and may be influenced by medication status, diet, inflammation, and other systemic factors.
**Genetic susceptibility**	Genetic findings involving dopaminergic regulators such as DAT1, COMT, and monoamine oxidase-related pathways suggest that altered dopamine handling may influence intracellular dopamine exposure and oxidative vulnerability.	[59,95,96]	Human genetic studies with mechanistic interpretation	ADHD-specific, but indirect	Genetic associations do not directly confirm oxidative mechanisms, and functional effects are variable across populations and study designs.
**DAT/VMAT2-related evidence**	Experimental and mechanistic studies indicate that altered dopamine reuptake or impaired vesicular sequestration may increase the cytosolic dopamine pool, which is the fraction most vulnerable to oxidation and reactive oxygen species generation.	[17,20,46,85]	Mechanistic studies, experimental models, and selected ADHD-relevant evidence	Partially ADHD-relevant	Much of this evidence derives from broader dopaminergic research rather than direct demonstration in human ADHD brain tissue or in vivo ADHD-specific models.
**Animal models**	ADHD-relevant animal models, including spontaneously hypertensive rats and developmental exposure models, show oxidative imbalance, altered dopaminergic transport dynamics, and partial normalization after antioxidant or	[2,9,55,63,76,81,84]	Experimental animal studies	ADHD-relevant, indirect	Animal models reproduce only selected behavioral and neurobiological aspects of ADHD and cannot fully reflect the heterogeneity of the human disorder.

	catecholaminergic interventions.				
**Neuroimaging correlates**	Neuroimaging studies in ADHD consistently report abnormalities in fronto-striatal and mesocorticolimbic circuits, including altered connectivity and dopamine transporter-related changes in regions where high dopamine turnover may increase oxidative vulnerability.	[42,45,47,48,50,53]	Human neuroimaging studies	ADHD-specific, but indirect	Neuroimaging cannot directly measure dopamine oxidation or reactive oxygen species and therefore supports anatomical and functional plausibility rather than biochemical confirmation.
**Neurodevelopment and plasticity**	Experimental and developmental studies suggest that redox imbalance may influence dendritic spine maturation, synaptic plasticity, and long-term circuit remodeling in dopamine-relevant regions during critical developmental periods.	[72,76,79,82,102]	Experimental developmental studies and conceptual integration	Partially ADHD-relevant	Direct evidence linking dopamine-derived oxidative stress to synaptic pruning or developmental remodeling specifically in human ADHD remains limited.
**Therapeutic implications**	Available evidence suggests that psychostimulants may improve signaling efficiency despite increasing dopamine availability, whereas antioxidants, micronutrients, ferroptosis-related strategies, and interventions targeting dopamine compartmentalization remain potential adjunctive or exploratory approaches.	[16,40,45,93-98]	Clinical studies, preclinical studies, and translational inference	Mixed, ranging from ADHD-specific to exploratory	Most redox-targeted therapeutic strategies remain preliminary in ADHD, and direct evidence for clinical efficacy against dopamine-derived oxidative mechanisms is still insufficient.
