# Supplementary material for: Dopamine-Derived Oxidative Stress in Attention-Deficit/Hyperactivity Disorder: A Narrative Review of Molecular Mechanisms, Neural Circuitry, and Therapeutic Implications
Source: Antioxidants (Basel). 2026 May 13;15(5):613. doi: 10.3390/antiox15050613 (PMC13203595; doi:10.3390/antiox15050613)
Supplement: Supplementary file 1 [file antioxidants-15-00613-s001.zip › antioxidants-4276430-TEX.zip!/Definitions/antioxidants-4276430-g001.pdf]

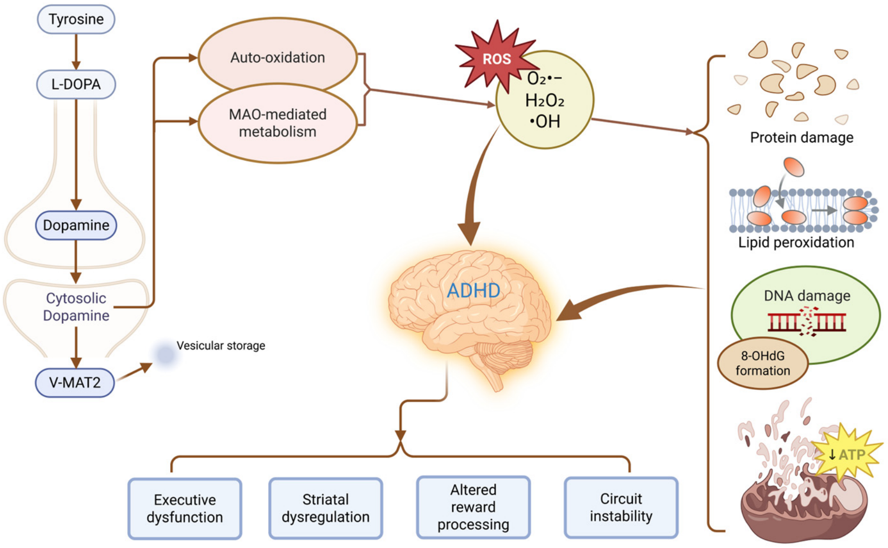

Tyrosine
L-DOPA
Dopamine
Cytosolic Dopamine
V-MAT2
Vesicular storage
Auto-oxidation
MAO-mediated metabolism
ROS
$O_2^{\bullet -}$
H_2O_2
•OH
ADHD
Protein damage
Lipid peroxidation
DNA damage
8-OHdG formation
↓ATP
Executive dysfunction
Striatal dysregulation
Altered reward processing
Circuit instability
