# Supplementary material for: Dopamine-Derived Oxidative Stress in Attention-Deficit/Hyperactivity Disorder: A Narrative Review of Molecular Mechanisms, Neural Circuitry, and Therapeutic Implications
Source: Antioxidants (Basel). 2026 May 13;15(5):613. doi: 10.3390/antiox15050613 (PMC13203595; doi:10.3390/antiox15050613)
Supplement: Supplementary file 1 [file antioxidants-15-00613-s001.zip › antioxidants-4276430-TEX.zip!/Definitions/antioxidants-4276430-g002.pdf]

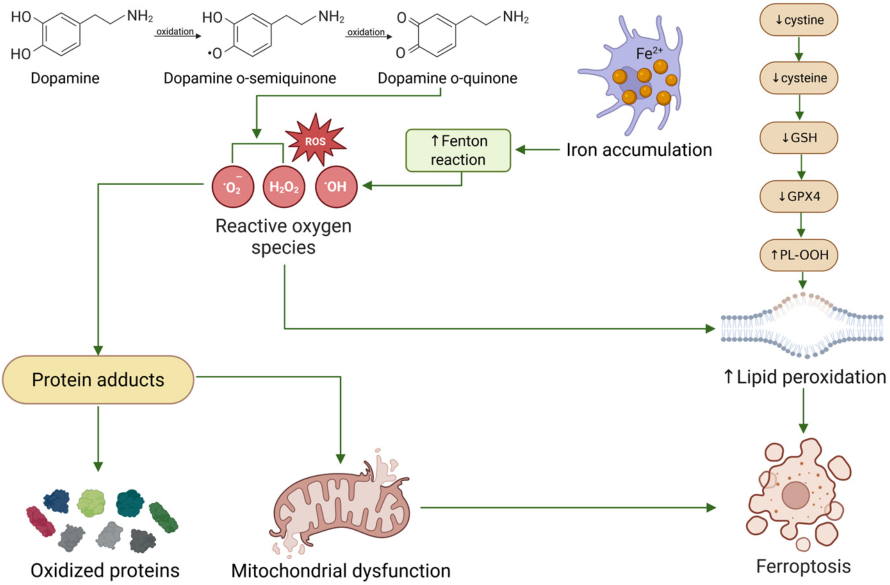

Dopamine
oxidation
Dopamine o-semiquinone
oxidation
Dopamine o-quinone
Fe2+
Iron accumulation
↑Fenton reaction
ROS
Reactive oxygen species
↓cystine
↓cysteine
↓GSH
↓GPX4
↑PL-OOH
↑Lipid peroxidation
Protein adducts
Oxidized proteins
Mitochondrial dysfunction
Ferroptosis
