# Supplementary material for: Dopamine-Derived Oxidative Stress in Attention-Deficit/Hyperactivity Disorder: A Narrative Review of Molecular Mechanisms, Neural Circuitry, and Therapeutic Implications
Source: Antioxidants (Basel). 2026 May 13;15(5):613. doi: 10.3390/antiox15050613 (PMC13203595; doi:10.3390/antiox15050613)
Supplement: Supplementary file 1 [file antioxidants-15-00613-s001.zip › antioxidants-4276430-TEX.zip!/Definitions/antioxidants-4276430-g003.pdf]

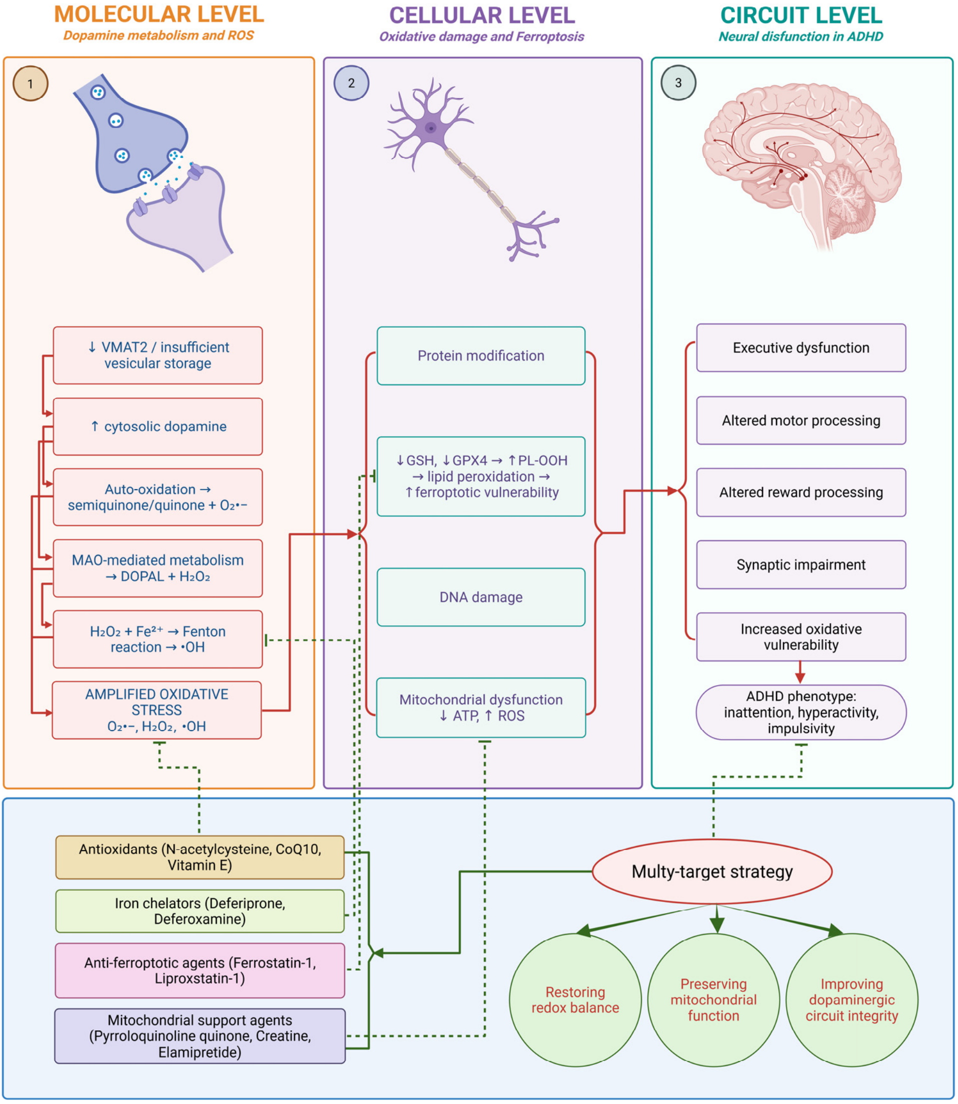
MOLECULAR LEVEL
Dopamine metabolism and ROS
CELLULAR LEVEL
Oxidative damage and Ferroptosis
CIRCUIT LEVEL
Neural disfunction in ADHD
1
2
3
↓ VMAT2 / insufficient vesicular storage
↑ cytosolic dopamine
Auto-oxidation → semiquinone/quinone + O₂•−
MAO-mediated metabolism → DOPAL + H₂O₂
H₂O₂ + Fe²⁺ → Fenton reaction → •OH
AMPLIFIED OXIDATIVE STRESS O₂•−, H₂O₂, •OH
Protein modification
↓GSH, ↓GPX4 → ↑PL-OOH → lipid peroxidation → ↑ferroptotic vulnerability
DNA damage
Mitochondrial dysfunction ↓ ATP, ↑ ROS
Executive dysfunction
Altered motor processing
Altered reward processing
Synaptic impairment
Increased oxidative vulnerability
ADHD phenotype: inattention, hyperactivity, impulsivity
Antioxidants (N-acetylcysteine, CoQ10, Vitamin E)
Iron chelators (Deferiprone, Deferoxamine)
Anti-ferroptotic agents (Ferrostatin-1, Liproxstatin-1)
Mitochondrial support agents (Pyrroloquinoline quinone, Creatine, Elamipretide)
Multy-target strategy
Restoring redox balance
Preserving mitochondrial function
Improving dopaminergic circuit integrity
